# Multi-omics in coronary atherosclerosis: mechanistic insights and advances in precision diagnosis and treatment

**DOI:** 10.3389/fendo.2026.1974206

**Published:** 2026-10-20

**Authors:** Jialong Chen, Fengsen Duan, Guizhen Zhu, Haiping Liu, Xiaoying Wang, Yueying Cui, Yuan Luo, Haitao Zhang

**Affiliations:** 1 Cardiovascular Department, Air Force Medical Center, Air Force Medical University, People's Liberation Army, Beijing, China; 2 Second Clinical Medical College, Anhui Medical University, Hefei, Anhui, China; 3 Laboratory of Clinical Medicine, Air Force Medical Center, Air Force Medical University, People's Liberation Army, Beijing, China

**Keywords:** biomarkers, coronary atherosclerosis, integrated multi-omics analysis, metabolomics, molecular mechanisms, proteomics

## Abstract

Coronary atherosclerosis, driven by the interplay of lipid metabolic derangements and chronic inflammation, constitutes the pathological basis of atherosclerotic cardiovascular disease and has emerged as the leading cause of mortality among Chinese residents. In patients with acute coronary syndrome, subsequent events may arise from initially nonculprit coronary lesions that appear angiographically mild. Invasive coronary angiography, the conventional diagnostic reference standard, is inherently invasive and incapable of characterizing plaque biological properties. Meanwhile, traditional risk factors only enable crude risk stratification, resulting in a critical shortage of precise early clinical screening modalities. Multi-omics technologies enable comprehensive molecular profiling of the human organism and effectively address the limitations of conventional detection approaches. Nevertheless, existing investigations remain largely confined to single-omics analyses; candidate biomarkers lack validation in large independent cohorts, and substantial bottlenecks persist in clinical translation. This review evaluates the respective advantages and limitations of genomics, epigenomics, transcriptomics, proteomics, metabolomics, lipidomics, and gut metagenomics in coronary atherosclerosis research. It elucidates the scientific value of integrated multi-omics analysis in dissecting pathogenic regulatory networks, unraveling gut-heart axis mechanisms, and identifying targets for precision therapy. Furthermore, it summarizes cutting-edge technological advances including single-cell multi-omics, spatial multi-omics, liquid biopsy, and AI-powered multi-omics integration, and compiles multi-omics risk prediction models developed on the basis of large-scale population cohorts. By bridging genetic susceptibility, cellular remodeling, peripheral molecular signatures, and plaque phenotypes, multi-omics integration can underpin early risk warning for high-risk populations, plaque subtyping, and therapeutic efficacy monitoring. Looking ahead, the establishment of standardized, multicenter prospective cohorts and the application of Mendelian randomization to infer causal relationships will be pivotal. These efforts are essential to translate multi-omics into a practical framework for comprehensive precision medicine across the entire continuum of coronary atherosclerosis.

## 1 Introduction

Cardiovascular diseases affect as many as 330 million individuals in China (1), and atherosclerotic cardiovascular disease (ASCVD) is the country’s leading cause of death. The disease typically develops insidiously: in the PROSPECT cohort of patients after acute coronary syndrome, nearly half of subsequent composite cardiovascular events were attributed to previously untreated nonculprit lesions, many of which appeared mild on baseline angiography; most such events were rehospitalizations for angina rather than MI or death (2). Plaque rupture remains an important mechanism of acute coronary events (3). Although coronary angiography is the gold standard for diagnosing coronary stenosis, it is invasive and carries risks such as access-site injury and contrast-induced nephropathy. Moreover, it assesses only the degree of luminal narrowing and cannot characterize plaque pathology or coronary microcirculatory injury. Traditional risk factors, including low-density lipoprotein cholesterol (LDL-C), hypertension, and diabetes, likewise provide only an approximate estimate of disease probability and cannot support precise, individualized prediction of disease onset. Current diagnostic and therapeutic approaches therefore remain insufficient for the early screening and diagnosis of coronary atherosclerosis (CA). This article is a narrative review. In this review, we summarize the latest research advances in CA multi-omics studies, discuss the core limitations of current research, and highlight the potential of integrated multi-omics profiling to identify novel diagnostic biomarkers, elucidate the molecular regulatory mechanisms of CA initiation and progression, and provide theoretical and technical support for early diagnosis, precision intervention and prognostic evaluation of CA, aiming to provide a comprehensive reference for future translational research in this field.

In this review, coronary atherosclerosis denotes atherosclerotic disease within the wall of the epicardial coronary arteries, documented pathologically or by coronary imaging or angiography. Coronary artery disease and coronary heart disease are retained only when they correspond to the population or outcome definition in the cited source and may include clinically recognized anatomical or ischemic coronary disease.

Omics technologies enable high-throughput, comprehensive molecular profiling of nucleic acids, proteins, and metabolites, thereby overcoming the limitations of conventional single-marker studies. Integrated multi-omics analysis can efficiently identify CA-specific biomarkers and elucidate the molecular mechanisms of disease progression, offering an important route to addressing current gaps in the early diagnosis of CA. However, multi-omics research in CA still has substantial limitations. Most studies remain confined to single-omics data mining and do not establish pathogenic causal regulatory networks through cross-omics integration; candidate biomarkers have rarely been validated in large prospective cohorts; clinically deployable translational models tailored to real-world settings are lacking; and omics data are often analyzed in isolation from coronary imaging and clinical phenotypes, precluding multidimensional, data-integrated risk-prediction systems. Accordingly, combined multi-omics profiling and integrative analysis can facilitate the discovery of novel CA biomarkers, clarify the molecular regulatory mechanisms underlying disease initiation and progression, and provide essential conceptual and technical foundations for early diagnosis, precision treatment, and prognostic assessment, with considerable clinical and scientific value.

Multi-omics can nominate cross-layer mechanisms and candidate biomarkers, but biological plausibility or improved discrimination in a development cohort does not establish clinical usefulness. Translation requires a locked and reproducible assay, validation in independent populations, assessment of calibration and incremental value over established clinical models, and prospective evidence that using the result changes decisions or improves outcomes.

## 2 Applications of single-omics approaches in CA research

### 2.1 Genomics and epigenomics

Genome-wide association studies (GWAS) analyzing large-scale population genetic data have identified hundreds of single-nucleotide polymorphisms significantly associated with coronary atherosclerosis, spanning pathways involved in lipid metabolism, inflammation, and cell proliferation. A major strength of genomics is its capacity for high-throughput, genome-wide variant screening, which can uncover previously unknown susceptibility loci. Because DNA remains unchanged throughout life, genomic markers are highly stable and have substantial value for early risk prediction (4). However, they indicate susceptibility rather than real-time disease progression, and the predominance of variants in non-coding regions often makes their specific pathogenic functions difficult to interpret directly (5, 6).

Whole-exome sequencing (WES) focuses primarily on rare variants within protein-coding regions and therefore provides an important complement to GWAS (4). Whereas GWAS predominantly identifies common variants, WES can detect rare, highly penetrant variants associated with atherosclerosis. In particular, it has successfully identified multiple pathogenic genes in families with familial hypercholesterolemia, thereby addressing an important limitation of GWAS. By restricting sequencing to coding regions, WES is substantially less costly than whole-genome sequencing and can precisely capture rare, high-effect pathogenic mutations. Its ability to explain complex, sporadic coronary heart disease driven by numerous small-effect polygenic variants is nevertheless limited (7).

Epigenomics examines changes in gene regulation without alteration of the DNA sequence, including DNA methylation and histone modifications (7). In human aortic tissue, atherosclerosis-associated hypermethylated regions frequently overlap aorta-associated enhancer chromatin and include smooth-muscle contractile genes; this association suggests, but does not by itself establish, effects on smooth-muscle phenotype (8). In coronary artery disease, blood 5-hydroxymethylation signatures have also been associated with clinical presentation (9). Histone methylation studies provide distinct evidence: H3K4me2 has been linked to MAP2K2 expression in carotid plaque (10), H3K9me2 modifies inflammatory responses in vascular smooth-muscle cells (11), and increased endothelial H3K27me3 can silence VE-cadherin and impair barrier function (12). H3K27me3 has also been implicated in endothelial-to-mesenchymal transition in experimental work (13), whereas myeloid Ezh2 deletion limits atherosclerosis in mice through immune-cell mechanisms (14). These tissue- and cell-specific results cannot be assumed to appear identically in peripheral blood or human coronary lesions (15, 16).

Evidence source qualification: Both endothelial cells and smooth muscle cells were studied using cell line models; The research results of Ezh2 are derived from a mouse model. Although the above studies support the relevant mechanisms in the mentioned tissues or models, without repeated validation in coronary artery tissues or cohorts with coronary artery phenotype characteristics, it cannot be concluded that these results are correlated with human coronary artery plaques.

### 2.2 Transcriptomics

Transcriptomic studies of atherosclerosis have interrogated endothelial cells, macrophages and vascular smooth muscle cells (VSMCs). In endothelial cells, N6-methyladenosine (m6A) modification alters epidermal growth factor receptor mRNA stability and contributes to endothelial dysfunction (16). In macrophages, desmosterol depletion promotes vascular inflammation and atherogenesis (17), whereas CITED2 restrains plaque formation by suppressing STAT1-IRF1 signaling (18). VSMC phenotypic switching from a contractile to a synthetic state is a central feature of disease. Lineage tracing integrated with single-cell RNA sequencing has identified a multipotent intermediate state derived from VSMCs that co-expresses stem-cell, endothelial-cell and monocyte marker genes and is therefore termed the SEM cells. During atherogenesis, VSMCs transition through this state before adopting diverse downstream fates; retinoic acid signaling is a key regulator of the transition (19). Transcriptomics provides a dynamic view of gene-expression states in coronary tissue and immune cells, enabling cell-resolved mapping of disease trajectories and activated pathways. Its limitations include the lability of RNA, which demands stringent sample handling, and the often non-linear relationship between transcript and protein abundance (20–22).

### 2.3 Proteomics

Proteomics has a central role in elucidating the molecular mechanisms of CA and identifying therapeutic targets, as it enables the precise detection and functional characterization of disease-associated differentially expressed proteins. Key biomarkers identified to date include lipoprotein-associated proteins (APOB and APOE), inflammatory proteins, and calcification-related proteins, providing a basis for CA risk stratification (20, 23–25). A proteomic and lipidomic analysis nested within the PACMAN-AMI trial identified plasma apolipoprotein and lipid changes after PCSK9-antibody treatment on a background of high-intensity statins (26). This can provide clues for exploring individualized treatment. Because proteins are the direct functional effectors of disease, proteomics can capture dynamic processes such as extracellular-matrix degradation and inflammatory responses during plaque formation and is considered the gold standard for identifying clinical biomarkers and therapeutic targets (4). Its major limitation is the exceptionally broad range of protein abundance in blood, from albumin to low-abundance cytokines. This analytical dynamic-range bottleneck can result in the omission of critical low-abundance proteins (7, 27–29).

### 2.4 Metabolomics and lipidomics

Recent studies have identified disturbances in amino-acid, lipid and microbial metabolism in coronary atherosclerosis. In patients undergoing elective coronary angiography, higher circulating trimethylamine N-oxide (TMAO) was associated with subsequent major adverse cardiovascular events (30). In a separate angiography cohort, imidazole propionate was associated with prevalent coronary artery disease; cell and mouse experiments linked it to endothelial dysfunction and increased atherosclerosis (31). Metabolite panels, including cholesterol sulfate and other lipid-related compounds, have shown diagnostic or prognostic promise in research cohorts, although their performance and clinical utility require independent prospective validation (20, 32–34). Metabolites reflect downstream physiological responses, and blood sampling permits repeated, minimally invasive measurement. Levels are also influenced by diet, medication, activity and sampling conditions, making standardization and confounding control essential (4, 32, 35).

Lipidomics characterizes molecular lipid species beyond conventional LDL-C measurement. In patients with established coronary artery disease, a score combining selected ceramides and phosphatidylcholines predicted residual cardiovascular event risk across independent cohorts (36). In experiments with reconstituted HDL, changing the sphingomyelin versus phosphatidylcholine composition altered cholesterol efflux and anti-inflammatory effects; this does not establish a validated clinical HDL ratio test (37). Lipid profiling can therefore refine risk hypotheses and reveal treatment-associated molecular changes, but the cited studies do not show that assigning lipid-lowering therapy by lipidomic profile reduces clinical events (23, 26, 36). Its limitations include closely related lipid isomers, differences in assay platforms, and confounding by lipid-modifying medications (4, 20, 23).

### 2.5 Gut metagenomics

Metagenomic studies report altered gut microbial composition and metabolic pathways in coronary artery disease, although geography, diet and medication can affect the associations (38, 39). Gut microbes convert dietary choline into trimethylamine, which host enzymes oxidize to trimethylamine N-oxide (TMAO); in patients undergoing angiography, higher TMAO was associated with later cardiovascular events (30). Experimental work links TMAO to vascular inflammation and platelet hyperreactivity, while the contribution of each pathway to individual human coronary plaques remains uncertain (40, 41). Imidazole propionate has been associated with prevalent coronary disease and impaired endothelial repair in cell and mouse models (31). Integrating microbial genes with measured metabolites can help test these pathways, but observational associations alone do not establish that changing the microbiome prevents coronary events (38, 42).

Each omics modality occupies a distinct functional niche with respect to the biological level interrogated, temporal resolution, relevance to clinical translation, and the scientific questions addressed. At the same time, every approach has characteristic strengths and limitations and is constrained by shared features and technical bottlenecks (**Table 1**).

**Table 1:** Comparative overview of individual omics approaches and their shared bottlenecks.

Omics approach	Biological level	Temporal characteristics	Proximity to the clinical phenotype	Core role in CA research/ key scientific question addressed
Genomics	DNA (sequence level)	Invariant throughout life (highly stable)	Most distal (indicates susceptibility)	Genetic-background screening: identify early susceptibility loci and calculate polygenic risk scores (PRSs).
Whole-exome sequencing	DNA (coding regions)	Invariant throughout life (highly stable)	Most distal (indicates susceptibility)	Rare variants: identify rare, high-effect pathogenic variants underlying conditions such as familial hyperlipidemia.
Epigenomics	DNA/histone modifications	Stage-dependent changes (reversible)	Relatively distal (mediates environmental interactions)	Environment-related regulation: determine how environmental factors such as smoking and high-fat diets stably silence or activate disease-associated genes.
Transcriptomics	RNA (expression intermediate)	Real-time dynamic changes (unstable)	Intermediate (reflects cellular state)	Pathway mapping: delineate signaling networks in vascular endothelial cells or immune cells within lesions.
Proteomics	Protein (functional effector)	Real-time dynamic changes (unstable)	Relatively proximal (reflects functional state)	Phenotypic execution: identify core functional proteins involved in CA formation and plaque rupture.
Metabolomics	Small-molecule metabolites	Rapid, transient changes (highly labile)	Most proximal (immediate pathological state)	Real-time diagnosis: capture transient molecular perturbations in blood associated with myocardial ischemia or plaque instability.
Lipidomics	Lipid molecular classes	Rapid, transient changes (readily affected by medication)	Most proximal (core lipid constituents)	Component-targeted profiling: precisely characterize specific atherogenic lipid subtypes beyond LDL-C and their links to foam-cell transformation.
Metagenomics	Gut microbial genes	Dynamically modulated by environment/diet	Indirect (distal regulation)	Gut-heart axis mechanisms: determine how microbial metabolites such as TMAO remotely exacerbate vascular inflammation.
Radiomics	Macroscopic tissue architecture	Slow, progressive evolution	Most proximal (direct anatomical phenotype)	Plaque identification: non-invasively assess the morphological features of vulnerable coronary plaques *in vivo* without tissue biopsy.

## 3 Applications of integrated multi-omics profiling and analysis in CA research

### 3.1 Integrated multi-omics profiling and analysis

Integrated multi-omics analysis has emerged as a core technological paradigm for elucidating the molecular mechanisms of CA and advancing precision diagnosis and treatment. The complementary strengths of cross-omics applications, spanning genes, proteins, metabolites, microorganisms, cells, spatial information, non-invasive liquid biopsy, and artificial intelligence, together establish a multilevel, multiscale systems-research framework (7, 20, 25) (**Figure 1**).

**Figure 1:**
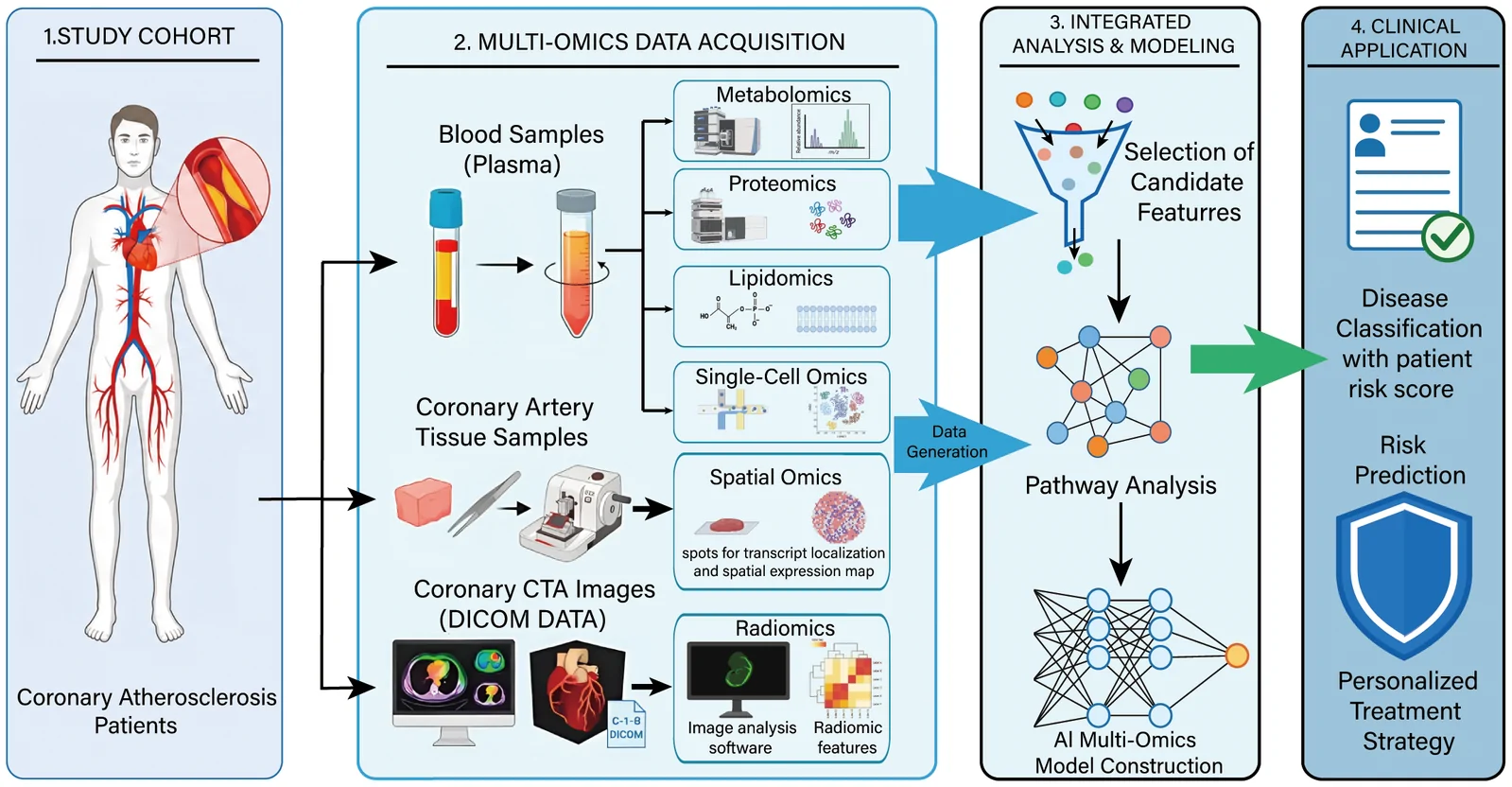
End-to-end workflow for integrated multi-omics assessment of coronary stenosis.

Metabolome-transcriptome integration can connect variation in gene expression with downstream metabolic patterns and nominate mechanisms for functional testing (7). In a coronary artery disease cohort, LPCAT1 haplotype 3 and several glycerophospholipid metabolites were associated with disease status (43). These findings implicate lipid remodeling as a candidate pathway, but that study did not establish an rs2043691-driven transcriptional effect or the proposed complete chain from LPCAT1 suppression to endothelial injury and coronary disease (**Figure 2**).

**Figure 2:**
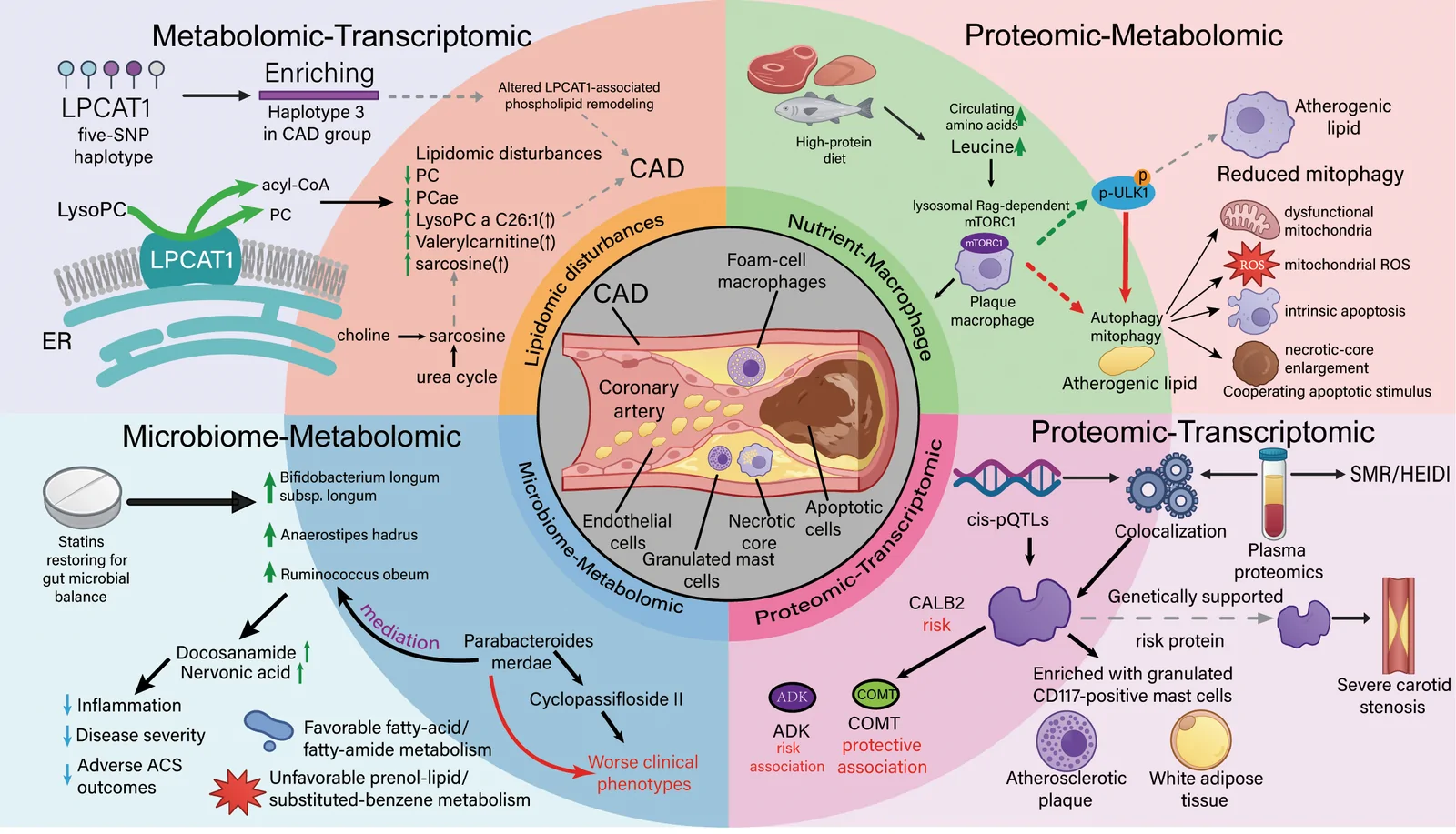
Schematic overview of multi-omics approaches for dissecting the mechanisms of coronary atherosclerosis initiation and progression.

Metabolome-microbiome integration centers on the “gut-heart axis.” By combining analysis of gut microbial composition, such as enrichment of Proteobacteria, with measurement of microbiota-associated metabolites, including TMAO, imidazole propionate, and 3-HB, this approach constructs microbiota-metabolite-host interaction networks (20, 31). It thereby delineates the pathways through which gut dysbiosis induces vascular inflammation and endothelial injury via microbial metabolites and provides a theoretical basis for microbiota-targeted intervention in CA. One study showed that the central pathway underlying statin treatment of acute coronary syndrome (ACS) operates through the gut-heart axis. Statins enriched anti-inflammatory or beneficial gut genera, including Roseburia and Blautia, which in turn remotely modulated host metabolism and markedly increased circulating levels of protective phospholipids such as phosphatidylserine. This network improved systemic homeostasis, attenuated chronic vascular endothelial inflammation, and ultimately significantly reduced the incidence of adverse cardiovascular events (44) (**Figure 2**).

Proteome-transcriptome integration interrogates regulatory uncoupling between gene transcription and protein translation. It can identify aberrant nodes at which transcription is increased but protein expression is suppressed, as well as key molecules that accumulate at the protein level despite unchanged transcription, thereby precisely locating core regulatory events in CA pathogenesis and providing direct evidence for the development of protein-targeted therapies (20, 23, 24). One study showed that mast cells exposed to an inflammatory microenvironment selectively upregulate CALB2 transcription and secretion, resulting in pathological increases in circulating and local CALB2 protein at the proteomic level. CALB2 perturbs calcium dynamics in macrophages, induces foam-cell formation, and erodes the fibrous cap of plaques, ultimately driving unstable atherosclerosis and severe vascular stenosis through a genetically supported causal mechanism (45) (**Figure 2**).

Proteome-metabolome integration enables bidirectional analysis of protein function and metabolic responses. On the one hand, it defines how differentially expressed proteins, including inflammatory mediators and matrix metalloproteinases, regulate metabolic pathways (23, 24); on the other, it reveals the feedback effects of metabolites on protein activity. This approach substantially improves the specificity and reliability of diagnostic and prognostic biomarkers and thereby clarifies the mechanisms through which protein abnormalities mediate metabolic imbalance (35). In a mouse atherosclerosis model, one study showed that a high-protein diet raises levels of amino acids such as leucine and activates mTORC1 in macrophages. This complex suppresses ULK1/ATG5-mediated mitophagy, causing damaged mitochondria and reactive oxygen species (ROS) to accumulate and triggering caspase-9-mediated intrinsic apoptosis. Extensive macrophage apoptosis enlarges the necrotic core of atherosclerotic plaques and exacerbates plaque progression (46) (**Figure 2**).

Evidence-source qualification: In Apoe-deficient mice, a high-protein diet increased circulating amino acids, activated macrophage mTORC1, suppressed mitophagy and increased lesional macrophage apoptosis and necrotic-core formation (46). This provides mechanistic evidence in a mouse aortic atherosclerosis model; it does not establish that dietary protein produces the same plaque response in human coronary arteries.

The principal value of integrated multi-omics is its ability to connect circulating molecular signatures, intraplaque cellular heterogeneity and lesion spatial organization into a coherent pathogenic continuum. Studies increasingly combine clinical phenotyping and coronary imaging with plasma, stool and plaque-tissue sampling for metabolomic, lipidomic and proteomic profiling. Talmor-Barkan et al. integrated serum metabolomics, gut microbiome profiles and clinical data, showing that microbial functional variation is associated with coronary artery disease risk through linked metabolites and underscoring the contribution of host-microbiome interactions to circulating metabolic abnormalities (47). Lv et al. related plasma metabolic signatures to subsequent cardiovascular events in coronary artery disease and identified molecular perturbations shared across, or specific to, distinct outcomes, providing a basis for patient stratification (48). Using GeoMx spatial transcriptomics on autopsy-derived human coronary arteries from 10 individuals with histologically classified stable or unstable plaques, Gastanadui et al. identified region- and cel- specific transcriptional programs while preserving plaque architecture. Unstable plaques exhibited enrichment of inflammatory and prothrombotic signaling, These findings associate spatially organized inflammatory and cellular-remodeling programs with coronary plaque instability (49). Cross-validation of tissue-derived signals against plasma proteomic and lipidomic markers may therefore identify candidates that are mechanistically linked to plaque biology yet measurable in peripheral blood.

### 3.2 Computational integration and validation of heterogeneous data

Early integration concatenates normalized features from matched participants before regularized or other supervised modelling; it is simple but can be dominated by a high-dimensional modality and may discard participants with incomplete assays. Intermediate integration learns shared and modality-specific latent factors or supervised components, as in MOFA and DIABLO, reducing dimension while retaining cross-layer structure (50, 51). Late integration combines modality-specific predictions and is useful when full assay coverage is unavailable, although it can miss feature-level interactions. Network-based integration, such as similarity network fusion, combines patient-similarity graphs estimated separately for each modality (52). The choice of framework should follow the clinical question, matched-sample availability, sample size, missingness and interpretability rather than presumed superiority of any algorithm.

Two design axes should be distinguished. Horizontal integration combines the same omics layer across cohorts, sites or batches after harmonization, principally to increase power and assess transportability, whereas vertical integration links different omics layers measured in the same participants. Within vertical integration, information may be combined by early data-level fusion, intermediate latent-factor or joint-representation learning, network-based integration, late decision-level fusion, or knowledge-guided mechanistic modelling. The integration stage is distinct from the downstream learner, which may be regression, a tree-based method, a neural network or a graph model. Mathew et al. compare horizontal and vertical integration and early, intermediate and late fusion, whereas Li et al. organize computational models into classical machine learning, deep representation learning, graph or network modelling and mechanistic systems-biology approaches (53, 54). Li et al. concerns immuno-oncology and is cited only for general computational principles, not as evidence of coronary clinical efficacy (**Table 2**).

**Table 2:** Comparison of computational strategies for multi-omics integration.

Strategy	Integration point	Suitable use	Strengths	Limitations and controls
Early or data-level fusion	Concatenate modality-specific normalized features before modelling	Matched, relatively complete datasets; parsimonious prediction after prespecified feature reduction	Simple; permits direct cross-layer interactions	Sensitive to scale and dimensional imbalance; may lose participants with missing assays; all preprocessing and tuning must remain inside training folds
Intermediate or representation-level fusion	Learn shared and modality-specific latent factors or embeddings	Subtype discovery and coordinated cross-layer signals	Reduces dimensionality while retaining shared and modality-specific structure	Factors may be difficult to interpret; small cohorts invite overfitting; report stability, hyperparameters and external reproducibility
Network-based integration	Link or fuse feature or patient-similarity networks	Pathway analysis, clustering and molecular-module prioritization	Uses relational structure and biological knowledge	Depends on network construction and prior knowledge; test alternative networks and validate biologically
Late or decision-level fusion	Combine predictions from separate modality-specific models	Partly missing modalities or heterogeneous platforms	Flexible; can retain participants without complete assay panels	May miss feature interactions; stacking must use out-of-fold predictions; recalibrate after fusion
Knowledge-guided or mechanistic integration	Constrain molecular data with pathways, causal graphs or mechanistic models	Target prioritization, hypothesis generation and intervention simulation	Improves interpretability and makes assumptions explicit	Depends on incomplete knowledge and assumptions; triangulate with genetic, experimental and prospective evidence

No integration strategy is universally superior. Selection should follow the clinical question, matched-sample availability, cohort size, dimensionality, missingness, platform heterogeneity and interpretability. All data-dependent preprocessing must be confined to the training data (53, 54).

A reproducible pipeline should harmonize patient identifiers, sampling times, endpoint definitions and units before modelling; perform modality-specific quality control and normalization; and record collection site, assay batch, medication and other potential confounders. Batch correction, including empirical-Bayes approaches where appropriate, must preserve biological signal and cannot rescue a design in which batch is completely confounded with case status (55). Missing modalities should be reported by patient and site and handled with a method that models incomplete blocks, a prespecified training-only imputation strategy, or modality-specific late fusion; complete-case and missingness-pattern sensitivity analyses remain necessary (50). High-dimensional discovery requires prespecified feature selection, false-discovery-rate control, and a locked model (56). Imputation, scaling, batch adjustment, feature selection and hyperparameter tuning must be confined to training folds within nested cross-validation; all samples from one participant must remain in the same fold, and an independent site or time-period test set should stay untouched. Report discrimination, calibration, uncertainty and incremental value over clinical predictors, as recommended for prediction-model reporting (57).

Discrimination should not be equated with clinical utility. The AUC describes ranking within a dataset but does not establish accurate predicted probabilities, an actionable threshold, or benefit from using the model. Evaluation should report confidence intervals, calibration plots, calibration intercept and slope, an overall accuracy measure such as the Brier score, and sensitivity, specificity and predictive values at prespecified clinically meaningful thresholds. A new omics model should be compared with the appropriate standard clinical model, with incremental value and decision-curve net benefit assessed relative to existing care, treat-all and treat-none strategies. Independent validation across institutions, time periods, assay platforms and relevant subgroups is required to assess transportability; a prospective impact or intervention study is needed before claiming that model-guided care improves decisions or outcomes (57, 58).

### 3.3 Scientific value of integrated multi-omics in the initiation and progression of CA

#### 3.3.1 Transition from single-dimensional analysis to systems biology

The pathobiology of CA involves multiple cell types, including endothelial cells, monocytes/macrophages, smooth muscle cells and T cells. Heterogeneity in plaque composition and morphology can culminate in plaque rupture or erosion and subsequent thrombosis (59). Because single-omics approaches interrogate individual cells, genes or metabolites in isolation, they cannot adequately capture the complexity and heterogeneity of CA. Integrated multi-omics overcomes this limitation by providing sequential, complementary molecular snapshots along a putative genotype-to-phenotype pathway that still requires causal testing: genomics defines inherited susceptibility; transcriptomics captures dynamic changes in gene expression (60); proteomics reports the state of functional execution (24); metabolomics captures the resulting physiological consequences (7); and microbiome profiling adds the emerging dimension of host-microbiota interactions (15).

#### 3.3.2 Causal inference and mechanistic elucidation

Integrated multi-omics can strengthen target prioritization by combining genetic instruments with measured proteins and metabolites. Mendelian randomization may support causal inference when its instrument-validity and other assumptions are met, but its results require triangulation with independent data and experiments (4, 61, 87). The gut TMAO pathway is a separate example of observational and experimental evidence, not a Mendelian-randomization result: circulating TMAO was associated with later cardiovascular events in an angiography cohort (30), and cell experiments showed MAPK/NF-κB-dependent vascular inflammatory responses (41). Mouse and human platelet studies also linked TMAO to platelet reactivity and thrombosis potential (40). These studies do not establish a complete TMAO-to-human-coronary-plaque-rupture causal chain.

Mendelian randomization (MR) depends on instrument relevance, independence from exposure-outcome confounders, and the exclusion restriction that the genetic variant affects the outcome only through the exposure. Horizontal pleiotropy can violate the last assumption; sensitivity analyses such as weighted-median estimation, MR-Egger and outlier checks should be compared, but concordant estimates do not prove that all instruments are valid. Weak instruments should be identified using instrument-strength statistics, while allele harmonization, ancestry-matched linkage-disequilibrium handling, possible sample overlap and population stratification must be addressed explicitly (62, 63).

For cis-eQTL or cis-pQTL analyses, colocalization or fine-mapping can test whether exposure and disease associations are consistent with a shared variant rather than separate signals in linkage disequilibrium; even a shared signal does not by itself establish mediation, druggability or clinical safety (62, 64). Tissue-specific expression and experimental perturbation should triangulate the genetic result before a target is prioritized for intervention.

#### 3.3.3 Precision target identification and dynamic monitoring of therapeutic responses

Integrating microbiome, metabolome, transcriptome and proteome data may improve the prioritization of candidate targets, provided that association findings are followed by perturbation experiments and independent replication (4, 65). For example, nonlethal inhibition of microbial trimethylamine production reduced TMAO and atherosclerosis in mice (66). NF-κB activation by TMAO has been demonstrated in vascular-cell experiments (41), but candidate interventions at these different levels remain at different stages of validation. The cited evidence does not measure shorter drug-development timelines or fewer clinical failures.

Intervention studies can use repeated omics measurements to quantify molecular responses to treatment. A nested PACMAN-AMI analysis profiled apolipoprotein and lipid changes after alirocumab or placebo on high-intensity statin therapy (26); the parent randomized trial measured coronary plaque changes by serial intravascular imaging (67). An observational acute coronary syndrome study also associated prior statin use with differences in gut microbiota and circulating metabolites (44). These studies support molecular monitoring as a research approach, but do not yet validate a multi-omics rule for identifying nonresponders, switching therapy or preventing adverse effects. Accordingly, classifying poor responders or nonresponders and changing therapy on that basis remain proposed future uses; they require prospectively specified thresholds, independent validation and an intervention study showing that model-guided care improves outcomes (57, 58).

## 4 Emerging multi-omics technologies

### 4.1 Spatial multi-omics

Spatial multi-omics have expanded spatial profiling of human coronary atherosclerosis. Campos et al. combined GeoMx regional profiling with CosMx single-cell spatial transcriptomics to map immune-cell distributions, transcriptional programs and cellular neighborhoods across different stages of coronary atherosclerosis (68). Bleckwehl et al. integrated a harmonized single-cell RNA-sequencing atlas with spatial transcriptomic data from 12 human arterial specimens, implicating ACKR1-positive microvascular endothelial cells in lymphocyte recruitment and mapping smooth-muscle-cell transition toward fibromyocytes (69). More recently, Amrute et al. combined single-cell RNA and surface-protein profiling, epitope mapping and spatial transcriptomics across 27 human coronary arteries, localizing FAP-positive modulated smooth muscle cells within macrophage-rich neointimal niches. Together, these studies demonstrate how spatial multi-omics can connect molecular cell states with plaque microarchitecture, although prospective validation of their prognostic and therapeutic relevance remains necessary (70).

### 4.2 Single-cell multi-omics

Single-cell and multimodal profiling reveals cellular heterogeneity and smooth-muscle-cell state transitions in atherosclerotic tissue (19, 71–75). Such tissue atlases can suggest possible cellular sources of circulating proteins, cell-free RNA and extracellular-vesicle cargo, but sequencing plaque cells alone cannot precisely trace the source of a blood-borne marker (25, 74–80). These methods are promising for biomarker discovery (81), but source attribution requires complementary evidence such as cell-specific secretion or extracellular-vesicle assays and, where feasible, lineage tracing.

The source tissue also differs across single-cell studies: Bashore et al. analyzed human carotid plaques (71); Traeuble et al. assembled an atlas spanning human carotid, coronary and femoral plaques (74); and Li et al. primarily profiled a time course in Apoe-deficient mouse aortic roots, with additional human coronary smooth-muscle-cell and CAD-genetic analyses (75). These results are complementary, but a cell state or pathway observed in another vascular bed or species requires confirmation in human coronary tissue before coronary-specific diagnostic or therapeutic claims are made.

Accordingly, the Bashore atlas is cited as human carotid-plaque evidence; the Traeuble atlas is described by its carotid, coronary and femoral components; and the Li trajectory study is described primarily as an Apoe-deficient mouse aortic-root time course with supporting human coronary smooth-muscle-cell and genetic analyses. No carotid-derived cell state is treated as a validated coronary biomarker without coronary-tissue replication.

### 4.3 AI-enabled multi-omics

Artificial intelligence can combine imaging and molecular measurements for research-stage classification and risk prediction. In a histology-referenced study, deep learning on hybrid IVUS-OCT images improved plaque-component classification compared with single-modality models and human readers (82). Deep-learning fusion of intravascular ultrasound (IVUS) and optical coherence tomography (OCT) leverages the complementary strengths of the two modalities to identify vulnerable plaques: the greater tissue penetration of IVUS permits quantification of deep lipid-core size and plaque burden, whereas the high spatial resolution of OCT enables assessment of fibrous-cap thickness and macrophage infiltration. A separate pilot study combined whole-blood transcriptomics with clinical data to predict coronary artery calcification (22). Neither study established that cardiac extracellular-vesicle multi-omics can diagnose myocardial ischemia, severe stenosis or future plaque rupture. Larger external and prospective validations are needed before these models guide treatment (83–85).

AI-based multi-omics and multimodal models are vulnerable to overfitting because the number of measured features often exceeds the number of participants. Optimistic performance can arise from information leakage when normalization, imputation, batch correction, feature selection or hyperparameter tuning is performed before partitioning; from placing repeated samples, vessels or image frames from one participant in both training and test sets; and from confounding by site, assay batch, medication, ancestry or imaging protocol. Class imbalance, missing modalities, unstable feature selection, limited interpretability and dataset shift further restrict transportability. All preprocessing and model selection should remain within the training data and, where appropriate, nested cross-validation; independent temporal or geographic cohorts should remain untouched until final evaluation. Studies should report uncertainty, calibration, prespecified threshold performance, incremental value, decision-curve net benefit and subgroup performance. Model and assay locking, transparent reporting, reproducible analysis, external validation and prospective impact studies are required before clinical deployment (57, 58).

### 4.4 Liquid biopsy

Blood-based proteomic, metabolomic, lipidomic and transcriptomic assays offer candidate markers for coronary disease and longer-term cardiovascular risk (20, 22, 24, 27, 36). For example, a pilot whole-blood transcriptomic model predicted coronary calcification (22), while plasma multi-omics models improved risk prediction in research cohorts (83). Extracellular vesicles are under investigation as another source of circulating markers (76, 77), These applications require standardized assays, source verification and prospective clinical testing. No cited study prospectively demonstrates population-level screening or molecular-subtype-guided treatment selection with a liquid-biopsy multi-omics signature; these applications remain investigational and require analytical standardization, external validation and clinical-utility testing (57, 58, 86).

In summary, integrated multi-omics strategies have deepened our understanding of coronary atherosclerosis across molecular, cellular, tissue and systemic circulatory scales. Metabolome-transcriptome and proteome-metabolome integration have uncovered cross-layer regulatory networks, microbiome-metabolome integration has illuminated emerging mechanisms along the gut-heart axis, and spatial and single-cell omics have resolved lesion heterogeneity and cellular origins. In particular, these approaches localize inflammatory responses, smooth muscle remodeling and determinants of plaque stability to specific microregions and map inflammatory signaling, smooth muscle cell phenotypic switching and immune-cell accumulation to sites such as the fibrous cap, necrotic core and regions surrounding microvessels. AI enables multimodal data integration and clinical translation; it can improve long-term risk stratification and support patient subtyping, target identification and therapeutic-response monitoring. AI and machine learning can further perform feature selection and network integration across omics data at different scales and dimensionalities. Luo et al. integrated proteomic, metabolomic and clinical variables using AI and demonstrated complementary predictive value across omics layers (83). Together, integrated multi-omics can establish a closed-loop framework spanning risk-factor discovery, mechanistic elucidation, vulnerable-plaque identification and prognostic prediction, while liquid biopsy extends this framework to non-invasive diagnosis. These strategies are complementary rather than interchangeable. Future studies should integrate additional multimodal data, including the epigenome, radiomics and clinical phenotypes, establish standardized multi-omics databases, and undertake validation in prospective clinical cohorts, ultimately enabling early risk detection, molecular subtyping, target discovery and individualized treatment of coronary heart disease and improving patient outcomes.

## 5 Clinical translation and future perspectives

Multi-omics integrates genomic, transcriptomic, proteomic, and metabolomic data to reconstruct the dynamic flow of information along the central dogma, determine whether molecular alterations propagate to the protein level and ultimately affect metabolic phenotypes, and account for nonlinear relationships between mRNA and protein abundance arising from post-transcriptional regulation, thereby nominating candidate regulators of phenotypic variation for functional validation (7, 20, 85). Multi-omics can dismantle disciplinary silos and identify key cross-omics nodes. Many regulatory processes underlying biological activity span multiple molecular dimensions; integrative analysis can identify drivers occupying central positions within these networks, which often represent optimal targets for disease intervention and drug development (83, 85, 87, 88). By integrating multidimensional data acquired at different time points and under different intervention conditions, multi-omics analysis can construct dynamic models of disease evolution to predict disease risk, progression, and prognosis. Some multi-omics models have improved risk discrimination in research cohorts, but generalizability depends on external validation and calibration (58, 83, 89). Repeated multi-omics sampling may eventually support dynamic monitoring; the cited studies do not yet establish that such models identify individual vulnerable plaques or improve outcomes when used to guide treatment (44, 74, 87).

## 6 Requirements for clinical implementation

Clinical translation requires an end-to-end evidence chain beyond biomarker association or model discrimination (**Table 3**). Pre-analytical protocols should prespecify specimen type, fasting and collection time, medication exposure, anticoagulant, processing delay, hemolysis, storage duration and freeze-thaw cycles. Analytical validation should establish accuracy, precision, detection limits, stability, lot-to-lot variation, and interlaboratory and cross-platform reproducibility; a locked targeted assay with calibrators and quality-control materials is generally more deployable than an untargeted discovery workflow. Batch correction must be prespecified, estimated without evaluation-set outcomes and supported by technical controls; it cannot recover biological effects when batch is confounded with disease status. Clinical use further requires repeatable and interpretable outputs, calibration in the intended population and assay-specific reference intervals or prospectively validated decision thresholds. The test should demonstrate incremental and net benefit beyond established risk scores, biomarkers and imaging. Turnaround time, infrastructure, data storage, total cost, equitable performance, reimbursement and health-economic value must also be evaluated. Reimbursement should follow evidence that a deployable assay changes management or improves outcomes, rather than high analytical performance alone (54, 57, 58).

**Table 3:** Qualitative clinical readiness of omics and imaging approaches for coronary applications.

Approach	Specimen and pre-analytical sensitivity	Standardization, reproducibility, turnaround and cost	External validation and current coronary use
Genomics/PRS	Blood or saliva DNA is stable; verify identity and ancestry.	Genotyping is mature; PRS construction and transfer across ancestries vary. Turnaround and cost exceed routine chemistry.	Risk models need calibration in the intended population; PRS is not a stand-alone coronary diagnostic test.
Whole-exome sequencing	Blood or saliva DNA is stable; variant quality and consent matter.	Sequencing is established, but variant interpretation adds time and cost beyond targeted testing.	Used for selected monogenic lipid disorders; not routine screening of sporadic coronary disease.
Epigenomics	Blood or vascular tissue; cell mixture, collection and storage affect signals.	Platforms and normalization differ; inter-laboratory reproducibility and practical turnaround remain application-specific.	Coronary methylation signatures are investigational; independent prospective validation is needed.
Transcriptomics	Whole blood or tissue; RNA degradation and cell composition require controlled handling.	Sequencing and qPCR workflows differ; high-dimensional signatures require a locked, repeatable assay.	A pilot whole-blood coronary-calcification model exists (22); no routine omics-based coronary test.
Proteomics	Plasma or serum; anticoagulant, hemolysis and freeze-thaw can affect concentrations.	Targeted assays may be reproducible, but broad panels require cross-platform harmonization and quality control; cost varies.	Individual proteins are used clinically; multi-protein coronary signatures remain investigational (27, 28).
Metabolomics	Plasma or serum; fasting, diet, timing and storage influence results.	Targeted assays are easier to standardize than untargeted features; annotation and platform drift add burden.	Candidate coronary panels have cohort-level evidence (20, 34); clinical decision benefit is unproven.
Lipidomics	Plasma or serum; medication, fasting, storage and lipid isomers matter.	Targeted species panels need reference materials and calibration; cost and turnaround depend on platform and batch size.	A ceramide/phospholipid score was validated in cohorts (36); routine treatment guidance is unproven.
Gut metagenomics	Stool; collection, transit, antibiotics and diet change profiles.	Extraction, sequencing and taxonomic pipelines differ; turnaround and cost exceed routine tests.	No validated coronary diagnostic or treatment-selection use.
Liquid biopsy/EVs	Plasma; platelet contamination, centrifugation and storage strongly affect vesicle yield and cargo.	Isolation and phenotyping require harmonization and reproducibility checks under MISEV2023 (86).	Exploratory coronary markers; no validated population screening or subtype-guided therapy.
Radiomics	CCTA, IVUS or OCT images; acquisition, reconstruction and segmentation affect features.	Imaging is used for selected indications, but algorithm reproducibility and costs depend on site and protocol.	Radiomic risk models require external validation and clinical-impact assessment (82, 90, 91).

**Table 3**: Reference intervals and decision thresholds are assay- and population-specific. Clinical readiness requires a locked assay, analytical validation, independent clinical validation, incremental-value and net-benefit analyses and, when management is guided by the result, a prospective clinical-impact study.

Models intended to identify asymptomatic high-risk individuals may facilitate earlier risk assessment only after transportability, calibration and clinical utility have been demonstrated in independent prospective populations (6, 92, 93). Multi-omics features can also be used to nominate potential drug targets, advancing precision medicine for the prevention and treatment of CA (23, 65, 76, 84). Although coronary angiography remains the “gold standard” among current diagnostic approaches, it is relatively insensitive to early or subclinical atherosclerosis and is costly. Blood molecular profiles may provide candidate features for earlier risk assessment, subject to prospective testing of clinical utility (7, 24, 27, 89). Single-omics and integrated multi-omics studies have yielded relevant diagnostic models (**Tables 4**, **5**).

**Table 4:** Verified prospective risk-prediction evidence organized by incident endpoint.

Model/role	Cohort and validation	Omics modality	Intended endpoint and verified result	Reference
Integrated germline and somatic genomic model	UK Biobank n=391,536; independent TOPMed validation n=34,177	Six germline/somatic genomic drivers, including genetically proxied proteomic and metabolomic scores	Incident CAD. UK Biobank C-statistic 0.734 for base+IGM and 0.750 for PCE+IGM; clinical-impact benefit was not tested.	(6)
Metabolic Vulnerability Index	UK Biobank n=171,242; no external cohort	nuclear magnetic resonance metabolomics	Incident ICD-10 I70 atherosclerosis, not coronary-specific stenosis; adjusted HR 1.22 per SD.	(34)
SCORE2 plus clinical, metabolomic and polygenic scores	UK Biobank n=297,463; 8,919 events; internal discovery/replication tranches	metabolomics	First-onset fatal/nonfatal CVD within 10 years, not coronary-only. C-index 0.743 vs 0.719 for SCORE2 (delta 0.024); no external population validation.	(89)
Random Forest classification model	Kangbuk Samsung Cohort n=42, comprising 14 patients with coronary artery disease (CAD) and 28 propensity score-matched (PSM) healthy controls.	shotgun metagenomics	achieving AUCs of 0.79 and 0.78 for bacterial species alone and predicted metabolites alone, respectively, which improved to 0.89 when both were combined, with key predictive features including inosine, alpha-muricholate, and F. prausnitzii. These findings suggest that integrating microbial taxonomic and metabolic features may enhance noninvasive risk stratification for CAD, but the small-sample results still require validation in larger cohorts.	(39)

Model-performance note: AUCs and C statistics are within-study results and should not be compared directly across rows because populations, sampling, prevalence, endpoints, reference standards, horizons and thresholds differ. Discrimination alone does not demonstrate calibration, incremental value, net clinical benefit or benefit from model-guided care. External validation denotes evaluation in participants independent of model development and tuning.

Inclusion criteria: adult prospective cohorts reporting an incident CAD, CVD or atherosclerosis endpoint, cohort size, molecular input and model association/discrimination. Clinical-only models are retained only as explicitly labelled benchmarks. Cross-sectional diagnostic, plaque-characterization and treatment-response studies are excluded from this table.

**Table 5:** Revised translational evidence grouped by clinical question; performance is study-specific and is not intended for cross-study ranking.

Clinical question	Study or model	Design, sample and validation	Data modality	Reference standard or endpoint	Within-study performance and evidence limit	Ref.
Detection of obstructive CAD	HART CADhs	Derivation n=636; internal validation n=275; external ED cohort n=241 with suspected MI	Three plasma proteins plus age, sex and prior PCI	Angiography; >=70% stenosis internally and >=50% externally	AUC 0.85 internally and 0.86 externally. The setting and endpoint threshold differed; calibration and clinical impact require intended-use evaluation.	(29)
Stenosis severity and obstructive CAD	GES12	Discovery n=110; training n=151; validation cohorts n=209 and n=206	Whole-blood 12-gene score; separate nomogram added clinical factors	Modified Gensini score, Sullivan Extent score and obstructive CAD are distinct outcomes	Validation AUCs 0.798/0.812 for Gensini, 0.776/0.778 for Sullivan and 0.834/0.792 for obstructive CAD. No impact trial.	(93)
Cross-sectional CAD classification	Trans-omics LPCAT1 model	Taiwanese cross-sectional cohort n=781; no independent external cohort	Clinical variables, targeted metabolomics and genome-wide SNPs	Study-group CAD definition included history, revascularization or >75% angiographic stenosis	AUC 0.917 and AUC-PR 0.74. Probabilities were overpredicted before recalibration; longitudinal and external validation are needed.	(43)
Coronary occurrence and angiographic severity	Plasma multi-omics and targeted metabolite panels	Single center: discovery n=246, temporal test n=93, blinded temporal validation n=104	Discovery used clinical variables, metabolomics and gut microbiota; final classifiers used metabolites	Angiography and Gensini categories 0, 1–40 and >40	Validation AUC 0.821-0.898 for occurrence and 0.649-0.849 for severity. Same-center temporal validation; not population screening.	(20)
Detection of myocardial ischemia	CCTA myocardial radiomics	Retrospective two-institution study, 101 patients/303 vessels; external-institution test set	CCTA radiomics plus conventional CCTA features	Dynamic CT-perfusion relative myocardial blood flow <0.8	Combined AUC 0.789 training and 0.726 test. Small vessel-level design and possible within-patient clustering; no prospective impact study.	(94)
Ex vivo plaque phenotype classification	Hybrid IVUS-OCT deep learning	Ten cadaveric human hearts; 992 training and 264 test frames	Multimodal intravascular imaging	Matched histology	Accuracy 86.7%, kappa 0.60 and 68% correct fibroatheroma classification. Frame-level ex vivo results are not prospective rupture prediction.	(82)
Treatment-related molecular change	PACMAN-AMI nested analysis	265 randomized-trial participants plus 34 registry patients	Plasma proteomics and lipidomics	Alirocumab versus placebo molecular changes; parent trial used serial plaque imaging	Group-level molecular response; no AUC, validated responder rule or model-guided treatment trial.	(26, 67)

Interpretive note: AUCs for anatomical stenosis, angiographic severity, cross-sectional CAD status, myocardial ischemia and other tasks must not be compared across rows. Frame-level or vessel-level metrics cannot be equated with participant-level clinical performance. Calibration, incremental value, decision-curve net benefit, external validation and prospective clinical impact are stated when available; their absence limits claims of clinical utility.

Evidence-source note for **Tables 4** and **5**: Human coronary imaging and angiography are direct coronary evidence; peripheral plasma, blood or stool from a coronary-phenotyped cohort are systemic evidence; cadaveric coronary tissue is ex vivo lesion evidence; and population-level composite outcomes, non-coronary vascular tissue, animal models or *in-vitro* systems support biological plausibility but do not directly validate human coronary-plaque mechanisms or clinical performance.

Clinical phenotypes are organized by the intended clinical question and its reference standard. Coronary atherosclerosis denotes the underlying arterial-wall disease; coronary artery disease denotes clinically or anatomically recognized coronary disease; and atherosclerotic cardiovascular disease is broader and also includes cerebrovascular and peripheral arterial events. Acute coronary syndrome is a clinical presentation, whereas myocardial infarction is an event defined by myocardial injury in an ischemic context. Obstructive coronary stenosis is an anatomical lumen endpoint, myocardial ischemia is a physiological perfusion endpoint, vulnerable plaque is a morphological or pathological phenotype, and major adverse cardiovascular events are prognostic composite outcomes. These endpoints are related but are not interchangeable. A model validated for one question should not be described as validated for another without a separate study using the appropriate reference standard.

Future efforts should focus on deeply integrating multidimensional data from genomics, transcriptomics, proteomics, metabolomics, epigenomics, and related modalities. Co-expression networks can generate testable cross-omics hypotheses, while MR can evaluate specific causal questions only under its instrument assumptions; neither approach alone establishes a disease-driving molecule or a safe therapeutic target (7, 62–64). Large, multicenter prospective cohorts should be established to systematically validate multi-omics-derived biomarkers and evaluate their predictive performance and stability in real-world clinical settings (20, 44). Standardization of sample processing, data acquisition, and bioinformatic analysis, together with the establishment of shared databases, will facilitate cross-study comparisons and data reuse (7, 85, 87).
